# A novel *LARGE1-AFF2* fusion expanding the molecular alterations associated with the methylation class of neuroepithelial tumors with *PATZ1* fusions

**DOI:** 10.1186/s40478-022-01317-8

**Published:** 2022-02-03

**Authors:** Arnault Tauziède-Espariat, Guillaume Chotard, François le Loarer, Jessica Baud, Rihab Azmani, Volodia Dangouloff-Ros, Nathalie Boddaert, Céline Icher-de-Bouyn, Edouard Gimbert, Lauren Hasty, Alice Métais, Fabrice Chrétien, Pascale Varlet, on behalf of the the RENOCLIP-LOC

**Affiliations:** 1 Department of Neuropathology, GHU Paris - Psychiatry and Neuroscience, Sainte-Anne Hospital, 1, rue Cabanis, 75014 Paris, France; 2 Institute of Psychiatry and Neurosciences of Paris (IPNP), Université de Paris, INSERM, U1266, 75014 Paris, France; 3 Department of Pathology, Groupe Hospitalier Pellegrin, CHU de Bordeaux, Bordeaux, France; 4 Université de Bordeaux, Talence, France; 5 Department of Biopathology, Institut Bergonié, Bordeaux, France; 6 INSERM U1218, ACTION, Institut Bergonié, Bordeaux, France; 7 Department of Pediatric Radiology, Hôpital Necker Enfants Malades, AP-HP, Université de Paris, INSERM U1163, Institut Imagine, Paris, France; 8 Department of Pediatric Oncology, Bordeaux University Hospital, Bordeaux, France; 9 Department of Pediatric Neurosurgery, Bordeaux University Hospital, Bordeaux, France

**Keywords:** LARGE1, AFF2, PATZ1, Neuroepithelial tumor

## Abstract

A novel DNA methylation class of tumor within the central nervous system, the "neuroepithelial tumor (NET), *PATZ1* fusion-positive” has recently been identified in the literature, characterized by *EWSR1*- and *MN1-PATZ1* fusions. The cellular origin of this tumor type remains unknown, wavering between glioneuronal or mesenchymal (as round cell sarcomas with *EWSR1-PATZ1* of the soft tissue). Because of the low number of reported cases, this tumor type will not be added to the 2021 World Health Organization Classification of Tumors of the Central Nervous System (CNS). Herein, we report one case of a CNS tumor classified by DNA methylation analysis as NET-*PATZ1* but harboring a novel *LARGE1-AFF2* fusion which has until now never been described in soft tissue or the CNS*.* We compare its clinical, histopathological, immunophenotypical, and genetic features with those previously described in NET-*PATZ1*. Interestingly, the current case presented histopathological (astroblastoma-like features, glioneuronal phenotype), clinical (with a favorable course), genetic (1p loss), and epigenetic (DNA-methylation profiling) similarities to previously reported cases of NET-*PATZ1*. Our results added data suggesting that different histomolecular tumor subtypes seem to be included within the methylation class “NET, *PATZ1* fusion-positive”, including non *PATZ1* fusions, and that further cases are needed to better characterize them.

**Supplementary Information:**

The online version contains supplementary material available at 10.1186/s40478-022-01317-8.

## Introduction

Neuroepithelial tumors (NET) with *PATZ1* fusions (NET-*PATZ1*) have been isolated by a distinct DNA methylation profile and are characterized by recurrent fusions of *PATZ1* in association with *EWSR1* or *MN1* genes [1]. These tumors present a wide variety of morphologies and immunophenotypes, having been initially classified as glioneuronal tumors, astroblastomas, ependymomas, glioblastomas, pleomorphic xanthoastrocytomas, primary neuroepithelial tumors, and round cell sarcomas, with different histopathological grades [2–11]. Because extra-CNS sarcomas may also harbor an *EWSR1-PATZ1* fusion and because of the uncertainty of the cellular origin of NET-*PATZ1*, this tumor type will not be added to the upcoming edition of the World Health Organization Classification of CNS Tumors [12]. Here we report a temporal tumor with a NET-*PATZ1* DNA methylation class (MC) but harboring a *LARGE1-AFF2* fusion. We compare its clinical, histopathological, immunophenotypical, genetic and epigenetic features with those previously described in NET-*PATZ1*.

## Case presentation

**Fig. 1:**
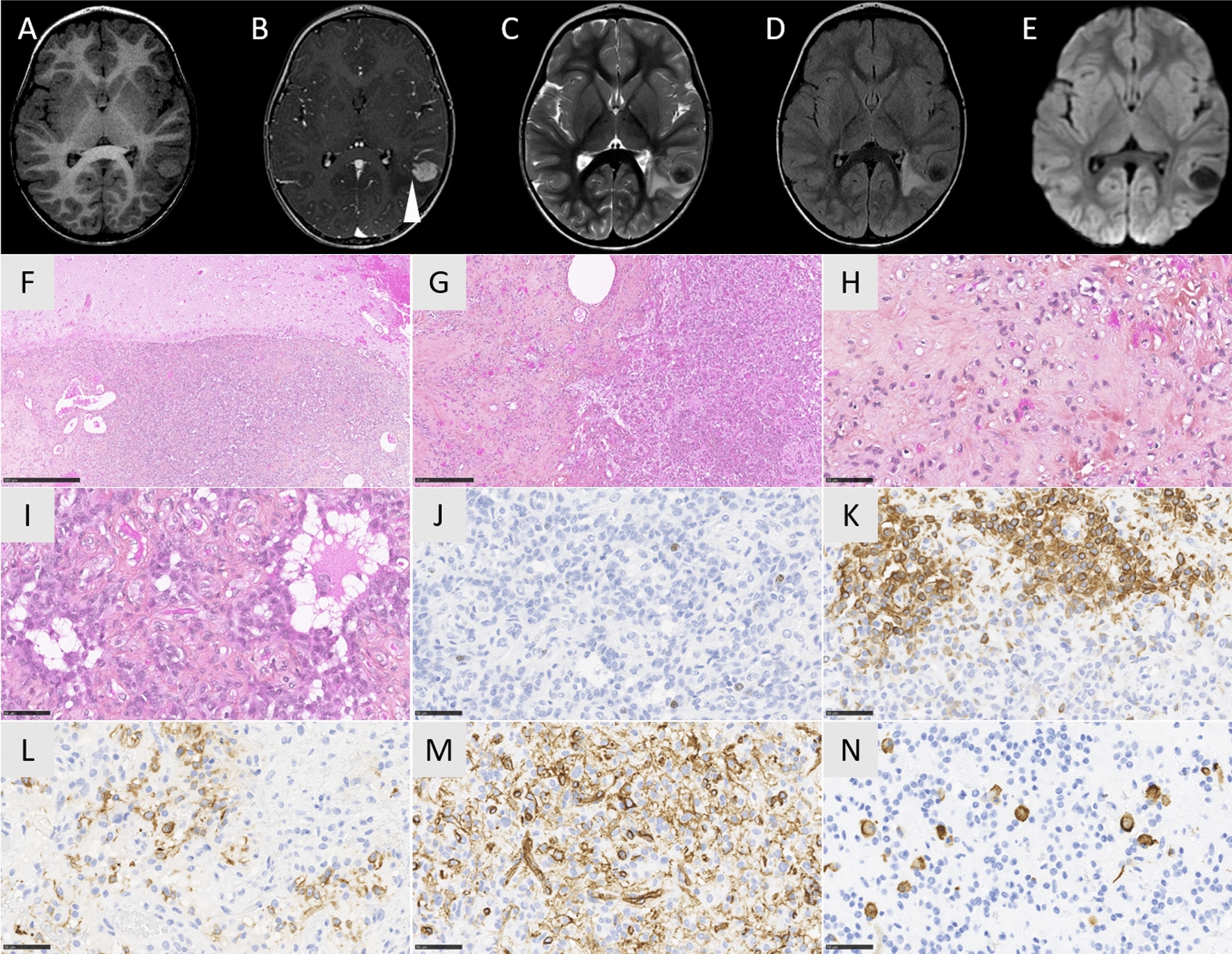
Radiological and histopathological features. **A** Axial T1-weighted magnetic resonance imaging image showing a left hyperintense temporal lesion. **B** Axial T1-weighted magnetic resonance imaging image after contrast injection showing an intense homogeneous enhancement of the lesion which is mainly solid with a small cyst (arrow). **C** Axial T2-weighted magnetic resonance imaging image showing an hypointensity of the lesion and a perilesional edema. **D** T2-FLAIR-weighted image showing the vasogenic perilesional edema. **E** Diffusion was not restricted. **F** The well delimitation of the tumor (HPS, magnification × 50). **G** The alternance of fibrous stroma containing few tumor cells and highest cellular areas (HPS, magnification × 200). **H** The collagenous stroma with few spindle cells (HPS, magnification × 400). **I** The cellular areas monomorphous cells with round to oval nuclei and abundant eosinophilic cytoplasm with microcystic changes (HPS, magnification × 400). **J** Low MIB-1 labeling index (magnification × 400). **K** GFAP immunoexpression by many tumor cells (magnification × 400). **L** MAP2 immunoexpression by a subset of tumor cells (magnification × 400). **M** Diffuse extravascular CD34 immunoexpression with CD34-positive ramified processes (magnification × 400). **N** Desmin immunoexpression by some tumor cells (magnification × 400). Black scale bars represent 500 µm for figure F, 250 µm for figure G, and 50 µm for figure H-N. HPS: Hematoxylin Phloxin Saffron.

**Fig. 2:**
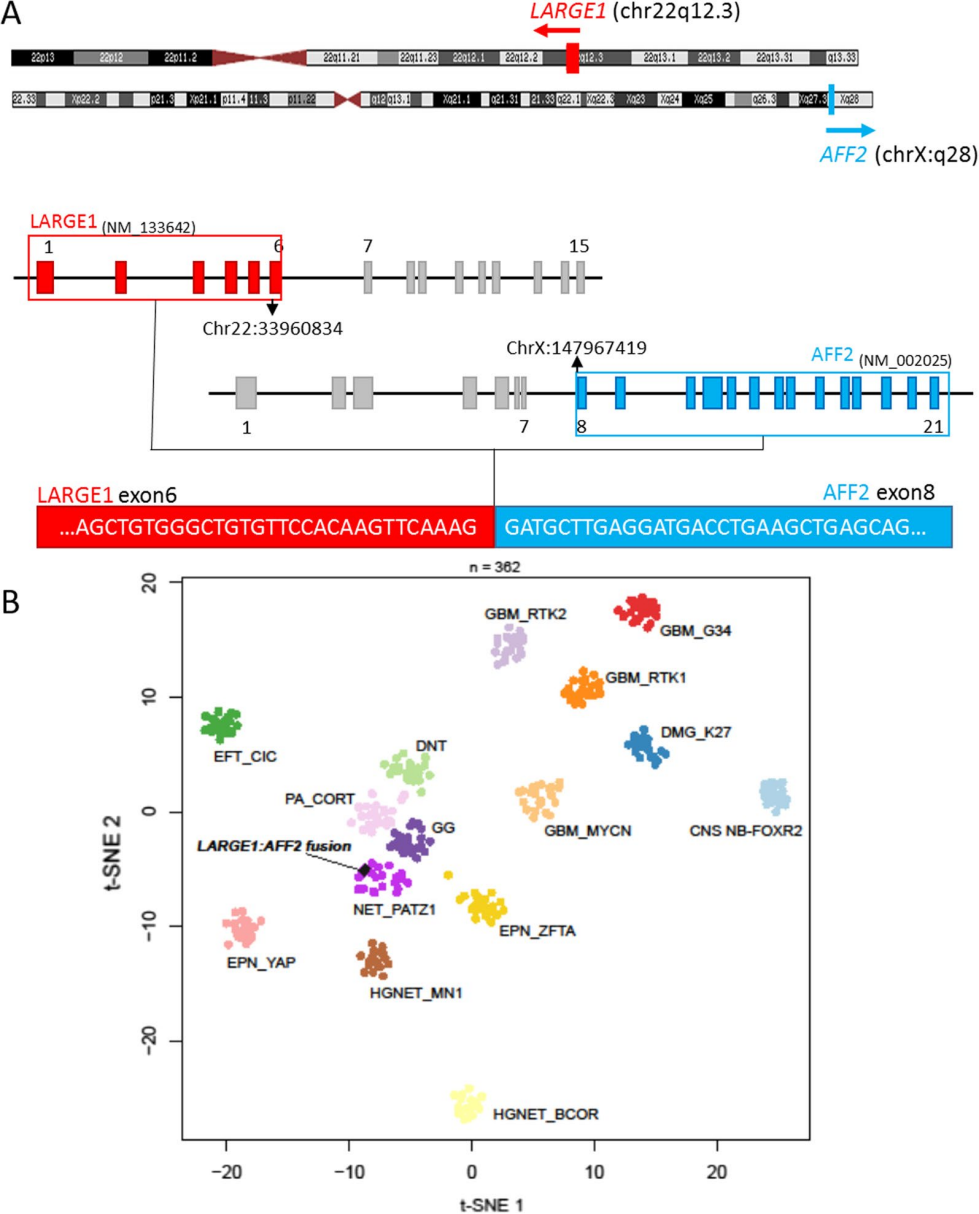
Illustration of the fusion and t-SNE analysis **A** RNAseq analysis highlights a fusion between *LARGE1* (red) and *AFF2* (blue) genes, respectively located on chr22q12.3 and chrX.q28 with a breakpoint in exon 6 for *LARGE1* and exon 8 for *AFF2*. **B** t-distributed stochastic neighbor embedding (t-SNE) analysis of the DNA methylation profile of the investigated tumor alongside 361 selected reference samples

A 3-year-old girl began experiencing seizures. Cerebral magnetic resonance imaging (MRI) showed a left temporal mass with a hyperintense signal on T1-weighted images, a hypointense signal on T2-weighted-images and with a homogeneous and intense contrast enhancement after gadolinium injection (Fig. 1A–D). The mass was cortical, well-circumscribed, solid with a small cyst, having slight perilesional edema and no leptomeningeal attachment. Neither hemorrhagic nor necrotic modification was observed and no calcifications were present on computerized tomodensitometry. Diffusion was not restricted (Fig. 1E). Gross total resection was achieved. Microscopically, the tumor was well-delineated from the brain parenchyma (Fig. 1F), and heterogeneous, presenting a sclerous stroma including isolated cells and nodules of glial cells with microcystic changes (Fig. 1G-I). No ependymal nor astroblastic pseudorosettes, or rhabdoid component were evidenced. The cytoplasm of the tumor cells was abundant and eosinophilic. Some tumor cells were plurinucleated. No mitotic figures, nor necrosis or microvascular proliferation were observed and the MIB-1 labeling index was low (around 5%) (Fig. 1J). Very few perivascular inflammatory infiltrates were present but no eosinophilic granular bodies or ganglion cells were observed. The tumor cells expressed glial markers (GFAP and Olig2 for a subset of cells; Fig. 1K), MAP2 (Fig. 1L) and NeuN (without expression of synaptophysin and chromogranin A), and CD34 (Fig. 1M). There was no immunopositivity for CKAE1/AE3, CK18, CD99, BCOR, SOX10, IDH1R132H or Lin28A. The tumor cells focally expressed desmin but there was no immunoreactivity for smooth muscle actin or myogenin (Fig. 1N). The expression of ATRX, BRG1, INI1 and H3K27me3 was retained. Because of the fibrous stroma, a diagnosis of astroblastoma was initially suggested but a FISH analysis of the *MN1* gene failed to reveal a rearrangement. RNA sequencing (including *EWSR1, MN1* and *PATZ1* genes) evidenced a *LARGE1-AFF2* gene fusion (Fig. 2A) and a DNA methylation analysis was conducted. The tumor was classified as NET-PATZ1 (with a strong calibrated score of 0.99) based on the DNA methylation profiling using a random forest machine learning classification algorithm as previously described (v12.3; Fig. 2B) [13]. Twelve months later, the patient is well without adjuvant treatment and no residual tumor on MRI.

## Discussion and conclusions

Here, we report an intracerebral tumor harboring a novel *LARGE1-AFF2* fusion, with clinical, radiological, histopathological, and epigenetic similarities to NET-*PATZ1.* Like most NET-*PATZ1*, our observation concerned a supratentorial tumor in a child (Table S1) [1–8, 11]. Whereas neuroradiological data of this recently described tumor type is scarce, our case presented as a solid and cystic lesion with T2-weighted hypointensity suggesting fibrotic content, well-circumscribed from the brain parenchyma, as previously reported [4, 8, 11]. NET-*PATZ1* encompassed a wide variety of morphologies in the literature, including glial, glioneuronal, embryonal tumors and sarcomas (Table S1). Based on the literature review and our case, the presence of a collagenous stroma and microcysts seem to be frequent in NET-*PATZ1* (Table S1) [1, 4, 8, 11]*.* Because of this pattern and because some of them present pseudorosettes (not seen in our case), pathologists tend to consider them a differential diagnosis for astroblastoma, *MN1*-fused [1, 2, 8]. However, NET-*PATZ1*, as with our case, exhibit frequent glioneuronal immunoprofiles and an extravascular expression of CD34 may be found, which is rare in astroblastomas (Table S1) [1, 11, 14]. The histopathological and epigenetic distinction between sarcomas with *EWSR1-PATZ1* fusion and NET, *PATZ1-*fusion positive is still not clear. Indeed, CNS and extra-CNS tumors with *PATZ1* fusion share some histopathological features (microcysts, collagenous stroma and pseudorosettes, and a mesenchymal component described in a part of NET-*PATZ1*) and a polyphenotypic immunoprofile (expression of glial, neuronal and CD34 in both) [1, 11, 15–17]. The DNA methylation analysis (v12.3) classified our case as a NET, *PATZ1*, although the tumor did not harbor a *PATZ1* fusion. Similarly, the case clustered with *PATZ1-*fused sarcomas, located outside the CNS on the whole RNA sequencing analysis. Our case presented a *LARGE1-AFF2* fusion which has not been previously reported in CNS or in soft tissue tumors. Whereas no *LARGE1* fusion has been described in tumors, several tumor types have been reported with *AFF2* fusions in association with different partners (*DEK* in squamous carcinomas, *RET* in lung cancer, *STAG2* in T-cell lymphoma) [18–21]. The fusion may drive the oncogenesis by deregulation of transcription as *AFF2* encodes a RNA-binding protein through the C-terminal domain that can activate transcription [22]. Moreover, the chimeric protein is predicted to contain the major functional domains of both LARGE1 and AFF2 proteins. Interestingly, *LARGE1* (22q12.3) and *PATZ1* (22q12.2) genes are found in close proximity on chromosome 22, and *PATZ1* was highly expressed at the RNA level (whereas *LARGE1* and *AFF2* are not) as observed in sarcomas with *PATZ1* fusion. Because DNA methylation profiles are thought to represent a combination of both somatically acquired DNA methylation changes and a signature reflecting the cell of origin [23], it is reasonable to assume that our case represents a subtype of NET-*PATZ1*. Because of the limited follow-up data and the heterogeneity of treatments applied in NET-*PATZ1* cases from the literature, no precise prognosis has been defined [1]. And despite histopathological signs of aggressivity, a probable intermediate grade has been suggested considering the better outcome associated with NET-*PATZ1* compared to high-grade tumors [1]. Our case is in line with these data, showing no recurrence one year after gross total resection without adjuvant treatment.

In conclusion, we expanded the defined MC NET-*PATZ1* genetic spectrum with one novel fusion that does not involve the *PATZ1* gene. This case illustrates that further studies are needed to characterize in detail this rare type of tumor in terms of cellular origin, histopathology, genetic features and outcome.

## Supplementary Information

**Additional file 1**. Table S1. Summary of clinical, histopathological and molecular data of CNS PATZ1-fused tumors reported in the literature
