# Supplementary material for: A novel LARGE1-AFF2 fusion expanding the molecular alterations associated with the methylation class of neuroepithelial tumors with PATZ1 fusions
Source: Acta Neuropathol Commun. 2022 Feb 3;10:15. doi: 10.1186/s40478-022-01317-8 (PMC8812055; doi:10.1186/s40478-022-01317-8)
Supplement: Supplementary file 1 — Additional file 1. Table S1. Summary of clinical, histopathological and molecular data of CNS PATZ1-fused tumors reported in the literature [file 40478_2022_1317_MOESM1_ESM.docx]

Table S1. Summary of clinical, histopathological and molecular data of CNS *PATZ1*-fused tumors reported in the literature

|  | **CNS tumors with *PATZ1* fusion (n=66)** |
| --- | --- |
| **Location** | Supratentorial (86%) >>> posterior fossa (7%) and spine |
| **Age** | Median age=11.5 YO (0;50) |
| **Sex ratio (F/M)** | 1.1 (36/32) |
| **Histopathology** | A wide variety of morphologies including HGG, EPN, AB, glioneuronal, PNET, PXA and sarcoma-like features  A recurrent pattern: solid tumor with perivascular pseudorosettes, hyalinized vessels and microcysts  Signs of aggressivity : high cellularity; median MIB1 labeling index 20% (0;70) ; necrosis (40%); microvascular proliferation (88%) |
| **Immunohistochemistry** | Frequent expression of glial markers (GFAP and Olig2)  Expression of one neuronal marker (synaptophysin or NeuN) in half cases  Extravascular expression of CD34 in less than half cases |
| **Fusion types** | EWSR1-PATZ1 (57%) and MN1-PATZ1 (43%) |
| **Outcome** | 33% recurrences (median PFS=14 months)  5% dead at the end of follow-up (median OS=26 months) |

AB: astroblastoma; EPN: ependymoma; F: female; HGG: high-grade glioma; M: male; OS: overall survival; PFS progression-free survival; PNET: primary neuroepithelial tumor; PXA: pleomorphic xanthoastrocytoma; YO years-old.
